# Circulating IL-6 and not its circulating signaling components sIL-6R and sgp130 demonstrate clinical significance in NSCLC patients treated with immune checkpoint inhibitors

**DOI:** 10.3389/fcell.2023.1324898

**Published:** 2024-01-16

**Authors:** Yoshiro Nakahara, Taku Kouro, Satoru Motoyama, Masatomo Miura, Kazuma Fujita, Yuka Igarashi, Naoko Higashijima, Norikazu Matsuo, Hidetomo Himuro, Feifei Wei, Shun Horaguchi, Kayoko Tsuji, Yasunobu Mano, Mitsuru Komahashi, Haruhiro Saito, Koichi Azuma, Tetsuro Sasada

**Affiliations:** ^1^ Department of Respiratory Medicine, Kanagawa Cancer Center, Yokohama, Kanagawa, Japan; ^2^ Department of Respiratory Medicine, Kitasato University School of Medicine, Sagamihara, Kanagawa, Japan; ^3^ Cancer Vaccine and Immunotherapy Center, Kanagawa Cancer Center, Yokohama, Kanagawa, Japan; ^4^ Division of Cancer Immunotherapy, Kanagawa Cancer Center Research Institute, Yokohama, Kanagawa, Japan; ^5^ Department of Comprehensive Cancer Control, Akita University Graduate School of Medicine, Akita, Japan; ^6^ Division of Esophageal Surgery, Akita University Hospital, Akita, Japan; ^7^ Department of Gastroenterological Surgery, Japanese Red Cross Akita Hospital, Akita, Japan; ^8^ Department of Pharmacy, Akita University Hospital, Akita, Japan; ^9^ Division of Respirology, Neurology, and Rheumatology, Department of Internal Medicine, Kurume University School of Medicine, Kurume, Fukuoka, Japan; ^10^ Department of Pediatric Surgery, Nihon University School of Medicine, Tokyo, Japan

**Keywords:** immune checkpoint inhibitor, non-small cell lung cancer (NSCLC), IL-6, soluble IL-6 receptor (sIL-6R), soluble glycoprotein 130 (sgp130), PD-1, PD-L1

## Abstract

**Introduction:** Clinical roles of plasma IL-6 levels have been reported in patients with various cancers, including non-small cell lung cancer (NSCLC), treated with immune checkpoint inhibitors (ICIs). However, the roles of other IL-6 signaling components, soluble IL-6 receptor (sIL-6R) and soluble gp130 (sgp130), in the plasma have not been elucidated.

**Methods:** Blood was collected from 106 patients with NSCLC before initiation of ICI treatment (anti-PD-1 or anti-PD-L1 antibody). Plasma levels of IL-6, sIL-6R, sgp130, and their complexes were assessed by Cox regression hazard model to evaluate their clinical significance. The clinical role of IL-6 or IL-6R genetic polymorphisms was also analyzed.

**Results:** Cox regression analysis showed that higher plasma IL-6 levels significantly predicted unfavorable overall survival (OS; hazard ratio [HR] 1.34, 95% confidence interval [CI] 1.05–1.68, *p* = 0.012) in NSCLC patients treated with ICIs. However, plasma sIL-6R and sgp130 levels showed no prognostic significance (*p* = 0.882 and *p* = 0.934, respectively). In addition, the estimated concentrations of binary IL-6:sIL-6R and ternary IL-6:sIL-6R:sgp130 complexes and their ratios (binary/ternary complex) were not significantly associated with OS (*p* = 0.647, *p* = 0.727, and *p* = 0.273, respectively). Furthermore, the genetic polymorphisms of IL-6 (−634G>C) and IL-6R (48892A>C) showed no clinical role by Kaplan-Meier survival analysis (*p* = 0.908 and *p* = 0.639, respectively).

**Discussion:** These findings demonstrated the clinical significance of plasma levels of IL-6, but not of other IL-6 signaling components, sIL-6R and sgp130, suggesting that classical IL-6 signaling, but not trans-signaling, may be related to anti-tumor immune responses in cancer patients treated with ICIs.

## 1 Introduction

Immune checkpoint inhibitors (ICIs), anti-PD-1 and anti-PD-L1 monoclonal antibodies (Abs), which enhance anti-tumor responses by suppressing immune inhibitory pathways in T cells, have demonstrated high clinical efficacy in several cancers, including non-small cell lung cancer (NSCLC) (Ribas and Wolchok, 2018; Grant et al., 2021). As a result, they have become the standard of care for patients with advanced and recurrent NSCLC. However, as response rates to these agents have been reported to be limited, patient selection should be recommended to avoid the possibility of serious immune-related adverse events (irAEs) and high costs (Postow et al., 2018; Johnson et al., 2022). In addition, it is important to identify the factors associated with clinical efficacy and mechanisms of action of ICIs to improve patient prognosis (Havel et al., 2019; Morad et al., 2021).

IL-6 is a multifunctional cytokine that regulates various aspects of the immune response and acute phase response (Hunter and Jones, 2015; Johnson et al., 2018; Jones and Jenkins, 2018). There have been several reports on the prognostic significance of plasma IL-6 in ICI-treated patients with various cancers, including NSCLC (Kang et al., 2020; Keegan et al., 2020; Laino et al., 2020; Kauffmann-Guerrero et al., 2021; Liu et al., 2022; Wang et al., 2022; Hu et al., 2023; Inoue et al., 2023). IL-6 has been reported to coordinate inflammatory responses in a context-dependent manner by triggering two distinct signaling modes, classical signaling and trans-signaling (Rose-John et al., 2006; Rose-John et al., 2023). In the classical signaling, IL-6 triggers signaling by binding to the membrane-bound IL-6 receptor (IL-6R) and glycoprotein 130 (gp130) expressed on specific cells, such as hepatocytes, monocytes, or macrophages. In contrast, in the trans-signaling, IL-6 binds to the soluble IL-6 receptor (sIL-6R) in the blood and forms binary IL-6:sIL-6R complexes that interact with gp130 expressed on almost all cell types regardless of IL-6R expression. However, they are inactivated by soluble gp130 (sgp130) through the formation of the ternary IL-6:sIL-6R:sgp130 complex (Garbers et al., 2011). Although the IL-6 trans-signaling via circulating sIL-6R and sgp130 has been reported to be associated with the risk or severity of inflammation-related diseases, such as cardiovascular events (CVE), metabolic syndrome, and COVID-19 infection (Weiss et al., 2013; Moreno Velasquez et al., 2015; Ritschel et al., 2016; Ziegler et al., 2019; Miri et al., 2021; Rodriguez-Hernandez et al., 2022; Li et al., 2023), no studies have been reported on the role of the IL-6 trans-signaling in cancer patients treated with ICIs.

It has been reported that IL-6 (−634G>C) and IL-6R (48892A>C) genetic polymorphisms are associated with the risk or prognosis in patients with some cancers (DeMichele et al., 2009; Motoyama et al., 2012; Stephens et al., 2012; Kibe et al., 2014; Ruzzo et al., 2014; Zhang et al., 2017; Gu et al., 2023). For example, we previously demonstrated that the IL-6 (−634G>C) genetic polymorphism showed a significant association with prognosis after surgery in advanced thoracic esophageal squamous cell carcinoma (Motoyama et al., 2012). In addition, we also reported that the IL-6R (48892A>C) genetic polymorphism was associated with prognosis in colorectal cancer patients undergoing cancer vaccination (Kibe et al., 2014). However, there are no reports on the role of these genetic polymorphisms in cancer patients treated with ICIs.

The aim of this study was to investigate the clinical roles of IL-6 signaling components, including IL-6, sIL-6R, sgp130, and their complexes in the plasma of NSCLC patients treated with ICIs. In addition, the effects of IL-6 (−634G>C) and IL-6R (48892A>C) genetic polymorphisms on their prognosis were also investigated.

## 2 Materials and methods

### 2.1 Patients

In this study, patients with advanced or recurrent NSCLC treated with anti-PD-1 Ab (pembrolizumab or nivolumab) or anti-PD-L1 Ab (atezolizumab) were enrolled at Kanagawa Cancer Center (Yokohama, Japan) or Kurume University (Kurume, Japan) between March 2017 and February 2021. This study was conducted in accordance with the provisions of the Declaration of Helsinki, and was approved by the Institutional Review Boards of Kurume University (approval numbers: 15210 and 19240) and Kanagawa Cancer Center (approval number: 2019-131) (Wei et al., 2023). Written informed consent was obtained from all participants prior to enrollment after the nature and possible consequences of this study were explained.

Patients received nivolumab (3 mg/kg of body weight or 240 mg, every 2 weeks), pembrolizumab (200 mg, every 3 weeks), or atezolizumab (1,200 mg, every 3 weeks) intravenously, with or without concurrent chemotherapy. Patients received treatment until intolerable toxicity or progressive disease (PD), as assessed by chest and abdominal computed tomography (CT) scans and cranial CT or magnetic resonance imaging (MRI) according to Response Evaluation Criteria in Solid Tumors (RECIST) version 1.1. PD-L1 expression in tumor cells was determined by immunohistochemistry (IHC) using anti-PD-L1 antibody (PD-L1 IHC 22C3 pharmDx kit; Agilent Technologies. Japan, Tokyo, Japan) in formalin-fixed paraffin-embedded tumor tissue sections.

### 2.2 Measurement of IL-6, sIL-6R, and sgp130 levels in the plasma

After enrollment, peripheral blood samples were collected in heparin-coated tubes from the patients before initiation of anti-PD-1 or anti-PD-L1 Ab treatment. Plasma was separated from whole blood by centrifugation and stored frozen until analysis. Plasma levels of IL-6, sIL-6R, and sgp130 were measured by the bead-based multiplex assay (Bio-Plex 200 system; Bio-Rad Laboratories, Hercules, CA) using 50-µl aliquots of 4-fold diluted plasma according to the manufacturer’s instructions.

### 2.3 Estimation of the IL-6 trans-signaling activation levels

The ratios of binary IL-6:sIL-6R and ternary IL-6:sIL-6R:sgp130 complexes, which were calculated from the molar concentrations of the IL-6, sIL-6R, and sgp130, were used as an index to estimate the level of IL-6 trans-signaling activation. Formulas described by Ziegler et al. (2019) (originally presented by Muller-Newen et al. (1998); Garbers et al. (2011)) were adopted for the calculation of binary and ternary complex concentrations. Briefly, the molar concentrations (mol/L) of IL-6, sIL-6R, and sgp130 were calculated by dividing the plasma concentration (ng/mL) by their respective molecular weights in kilodaltons (kD: IL-6, 23.7; sIL-6R, 50; sgp130, 100). The concentrations (nmol/L) of the binary and ternary complexes were estimated by using the following formulas.

$$\left[\text{IL}‐6:\text{sIL}‐6R\right]=0.5\left[\text{sIL}‐6R\right]+0.5\left[\text{IL}‐6\right]+0.5K_{D1}-0.5{\left({\left[\text{sIL}‐6R\right]}^{2}+{\left[\text{IL}‐6\right]}^{2}+2\left[\text{IL}‐6\right]K_{D1}+{K_{D1}}^{2}\right)}^{0.5}$$

$$\left[\text{IL}‐6:\text{sIL}‐6R:\text{sgp}130\right]=0.5\left[\text{sgp}130\right]+0.5\left[\text{IL}‐6:\text{sIL}‐6R\right]+0.5K_{D2}-0.5{\left({\left[\text{sgp}130\right]}^{2}+{\left[\text{IL}‐6:\text{sIL}‐6R\right]}^{2}+2\left[\text{IL}‐6:\text{sIL}-6R\right]K_{D2}+{K_{D2}}^{2}\right)}^{0.5}$$

[IL-6], [sIL-6R], and [sgp130] were replaced by the respective nmol/L concentrations of each factor in the plasma. K_D1_ and K_D2_ represent the dissociation constants for the binary and ternary complexes, 0.5 and 0.05 nmol/L, respectively (Muller-Newen et al., 1998). The binary/ternary complex ratio was calculated by dividing the concentration of the binary complex (nmol/L) by that of the ternary complex (nmol/L).

### 2.4 Analysis of IL-6 and IL-6R genetic polymorphisms

Peripheral blood mononuclear cells (PBMCs) were purified from peripheral blood by Ficoll-Paque Plus (GE Healthcare, Uppsala, Sweden) density centrifugation. DNA was extracted from PBMCs using a QIAamp Blood Kit (Qiagen, Hilden, Germany) and stored at −80°C until analysis. To investigate the IL-6 -634G>C (rs1800796) and IL-6R 48892A>C (rs2228145, Asp358Ala) genetic polymorphisms with the extracted DNA, genotyping was performed using the polymerase chain reaction-restriction fragment length polymorphism method, as previously reported (Motoyama et al., 2012). The following primers were used for amplification: forward 5′-GAG​ACG​CCT​TGA​AGT​AAC​TG-3′ and reverse 5′-AAC​CAA​AGA​TGT​TCT​GAA​CTG​A-3′ for IL-6 -634G>C (rs1800796) and forward 5′-CCT​TTG​AGG​CTT​TTG​ACA​G-3′ and reverse 5′-ACC​CAT​CTC​ACC​TCA​GAA​CAA-3′ for IL-6R 48892A>C (rs2228145).

### 2.5 Statistical analysis

Overall survival (OS) was defined as the period from the date of the first dose to the date of death from any cause or the date of censoring at the last follow-up examination. A Cox regression model was used to evaluate the significance of plasma IL-6 signaling components and clinicopathologic factors. Data for IL-6, sIL-6R, and sgp130 levels were normalized by subtracting the mean and dividing by the standard deviation (SD). Kaplan-Meier plots of OS were also used to demonstrate the clinical significance of plasma IL-6 levels or IL-6 and IL-6R genetic polymorphisms, and intergroup comparisons were assessed using the log-rank test. Optimal cut-off values for IL-6 were determined using the Cutoff Finder web application (https://molpathoheidelberg.shinyapps.io/CutoffFinder_v1) developed by Budczies et al., 2012. The optimal cut-off was defined as the point with the most significant separation by a log-rank test. Plasma IL-6, sIL-6R, and sgp130 levels were compared by Student’s t-test between the subgroups stratified by the IL-6 or IL-6R genetic polymorphism. *p* values of <0.05 were considered to be statistically significant. All statistical analyses were performed using JMP version 11 (SAS Institute Inc., Cary, NC).

## 3 Results

### 3.1 Patient characteristics

A total of 106 patients with NSCLC treated with anti-PD-1 Ab (pembrolizumab or nivolumab) or anti-PD-L1 Ab (atezolizumab) were enrolled between March 2017 and February 2021 (Table 1). The median age was 69 years (range, 43–96 years). Of the 106 patients, 79 (74.5%) were male and 27 (25.5%) were female; 91 (85.8%) had a good PS (Eastern Cooperative Oncology Group [ECOG] 0 or 1); 84 (79.2%) were current or former smokers; 71 (67.0%) and 35 (33.0%) had non-squamous and squamous cell carcinoma, respectively; 19 (17.9%) had EGFR, ALK, or ROS1 mutation/rearrangement. Among 90 patients with available tissue samples, the PD-L1 expression was absent or weakly positive (0%–49% of tumor cells) and strongly positive (>50% of tumor cells) in 56 (62.2%) and 34 (37.8%) patients, respectively. For treatment, pembrolizumab, nivolumab, and atezolizumab were used in 64 (60.4%), 24 (22.6%), and 18 (17.0%) patients, respectively, and chemotherapeutic agents were combined in 51 (48.1%) patients. Anti-PD-1 or anti-PD-L1 Ab was administered as the first-line and second-line or subsequent treatment in 57 (53.8%) and 49 (46.2%) patients, respectively. Among the 104 patients evaluated according to RECIST criteria, best overall responses of partial response, stable disease, and PD were observed in 31 (29.2%), 37 (34.9%), and 36 (34.0%) patients, respectively.

**TABLE 1:** Clinical characteristics and their association with OS in NSCLC patients treated with ICI.

Patient characteristics	Median (SD) or Number (%)	Univariate		Multivariate	
		HR (95%CI)	*p*-Value	HR (95%CI)	*p*-Value
Age (years), Median (SD)	69 (9.8)	1.01 (0.99–1.04)	0.330		
Sex, N (%)					
Male	79 (74.5%)	1		1	
Female	27 (25.5%)	1.94 (1.15–3.18)	0.014	2.10 (1.01–4.19)	0.048
PS, N (%)					
0–1	91 (85.8%)	1		1	
2–3	15 (14.2%)	2.52 (1.31–4.51)	0.007	2.04 (1.02–3.84)	0.045
Smoking, N (%)					
Ever	84 (79.2%)	1		1	
Never	22 (20.8%)	0.58 (0.35–1.03)	0.062	1.13 (0.53–2.48)	0.756
Histology, N (%)					
Non-Squamous	71 (67.0%)	1			
Squamous	35 (33.0%)	0.93 (0.54–1.53)	0.771		
Driver mutation, N (%)					
Wild type	87 (82.1%)	1			
EGFR, ALK, ROS1	19 (17.9%)	0.99 (0.52–1.76)	0.971		
PD-L1 expression, N (%)					
0%–49%	56 (52.8%)	1			
50%–100%	34 (32.1%)	0.80 (0.45–1.38)	0.426		
NA	16 (15.1%)				
PD-1/PD-L1 inhibitor, N (%)					
Pembrolizumab	64 (60.4%)	1		1	
Nivolumab	24 (22.6%)	0.72 (0.42–1.28)	0.254	0.90 (0.43–1.89)	0.790
Atezolizumab	18 (17.0%)	0.36 (0.15–0.82)	0.013	0.48 (0.18–1.15)	0.101
Combination chemotherapy, N (%)					
(−)	55 (51.9%)	1		1	
(+)	51 (48.1%)	0.63 (0.38–1.01)	0.056	0.94 (0.50–1.83)	0.862
Treatment line, N (%)					
1	57 (53.8%)	1		1	
>2	49 (46.2%)	1.58 (0.98–2.56)	0.061	1.58 (0.84–2.95)	0.155
IL-6 (pg/mL), Median (SD)	15.3 (33.9)	1.26 (0.98–1.56)	0.053	1.34 (1.05–1.68)	0.012
sIL-6R (pg/mL), Median (SD)	30927.7 (11303.1)	0.98 (0.76–1.26)	0.882		
sgp130 (pg/mL), Median (SD)	151388.7 (49732.4)	0.99 (0.77–1.26)	0.934		

Categorical variables are presented as the distribution of corresponding patient numbers. Continuous variables are presented as median and standard deviation (SD) values. Univariate and multivariate analysis was performed using the Cox proportional hazards model for overall survival (OS). Abbreviations: hazard ratio (HR), confidence interval (CI), performance status (PS), epidermal growth factor receptor (EGFR), anaplastic lymphoma kinase (ALK), not assessed (NA), soluble IL-6R (sIL-6R), soluble gp130 (sgp130).

### 3.2 Clinical significance of plasma IL-6 levels in non-small cell lung cancer patients treated with ICIs

The clinical significance of plasma IL-6 levels and other clinicopathologic factors before ICI treatment was evaluated in 106 patients with NSCLC using a Cox proportional hazards regression model (Table 1). Univariate analysis showed that sex, PS, smoking history, ICI type, combination chemotherapy, treatment line, and pre-treatment plasma IL-6 level tended to be significantly associated with OS (*p* = 0.014, *p* = 0.007, *p* = 0.062, *p* = 0.013, *p* = 0.056, *p* = 0.061, and *p* = 0.053, respectively). In addition, multivariate Cox regression analysis was further performed to evaluate the influence of these factors, which tended to be associated with OS in univariate analysis (*p* < 0.1). As shown in Table 1, sex, PS, and plasma IL-6 level were significantly associated with OS (hazard ratio [HR] 2.10, 95% confidence interval [CI] 1.01–4.19, *p* = 0.048; HR 2.04, 95%CI 1.02–3.84, *p* = 0.045; HR 1.34, 95% CI 1.05–1.68, *p* = 0.012; respectively).

**FIGURE 1:**
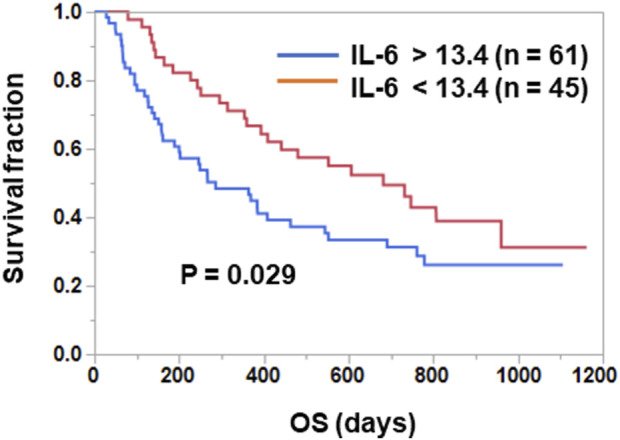
Prognostic significance of plasma IL-6 levels in ICI-treated NSCLC patients. Patients treated with ICIs were divided into two groups based on the plasma IL-6 levels. The optimal cut-off value for OS (13.4 pg/mL) was determined using the Cutoff Finder web application. Curves for OS were estimated using the Kaplan-Meier method and statistically evaluated using the log-rank test (*p* = 0.029).

Patients were divided into two groups according to their pre-treatment plasma IL-6 levels, and OS was compared between the two groups. As shown in Figure 1, OS was significantly longer in the IL-6^low^ group than in the IL-6^high^ group (median time, low 682 days vs. high 286 days; *p* = 0.029 [log-rank test]).

### 3.3 Clinical significance of plasma sIL-6R or sgp130 levels and the ratios of binary IL-6:sIL-6R and ternary IL-6:sIL-6R:sgp130 complexes in non-small cell lung cancer patients treated with ICIs

The clinical significance of plasma sIL-6R and sgp130 levels before ICI treatment was also evaluated in 106 NSCLC patients using a Cox proportional hazards regression model (Table 1). In contrast to IL-6, plasma sIL-6R and sgp130 levels did not show significant associations with OS by univariate analysis (*p* = 0.882 and *p* = 0.934, respectively). It has been reported that the binary complex composed of IL-6 and sIL-6R induces a pro-inflammatory response via the IL-6 trans-signaling, whereas they are inactivated by sgp130 through the formation of the ternary IL-6:sIL-6R:sgp130 complex (Rose-John et al., 2006; Garbers et al., 2011; Rose-John et al., 2023). Therefore, the concentrations of binary IL-6:sIL-6R and ternary IL-6:sIL-6R:sgp130 complexes as well as the ratio between them (binary/ternary complex) were evaluated for association with OS. As shown in Table 2, the binary IL-6:sIL-6R complex, ternary IL-6:sIL-6R:sgp130 complex, or their ratio did not show a significant association with OS (*p* = 0.647, *p* = 0.727, and *p* = 0.273, respectively).

**TABLE 2:** Clinical significance of binary IL-6:sIL-6R complex, ternary IL-6:sIL-6R:sgp130 complex, and the ratio between binary and ternary complexes in NSCLC patients treated with ICI.

Patient characteristics	Median (SD)	HR (95%CI)	*p*-Value
Binary IL-6:sIL-6R complex	0.162 (0.031)	0.16 (0.00–688.48)	0.647
Ternary IL-6:sIL-6R:sgp130 complex	0.099 (0.016)	0.08 (0.00–556909.2)	0.727
Binary/Ternary complex ratio	1.644 (0.100)	0.21 (0.01–3.70)	0.273

Univariate analyses were performed using a Cox proportional hazards model for overall survival (OS).

Abbreviations: standard deviation (SD), hazard ratio (HR), confidence interval (CI), soluble IL-6R (sIL-6R), soluble gp130 (sgp130).

### 3.4 Clinical significance of IL-6 (−634G>C) and IL-6R (48892A>C) genetic polymorphisms in non-small cell lung cancer patients treated with ICIs

**FIGURE 2:**
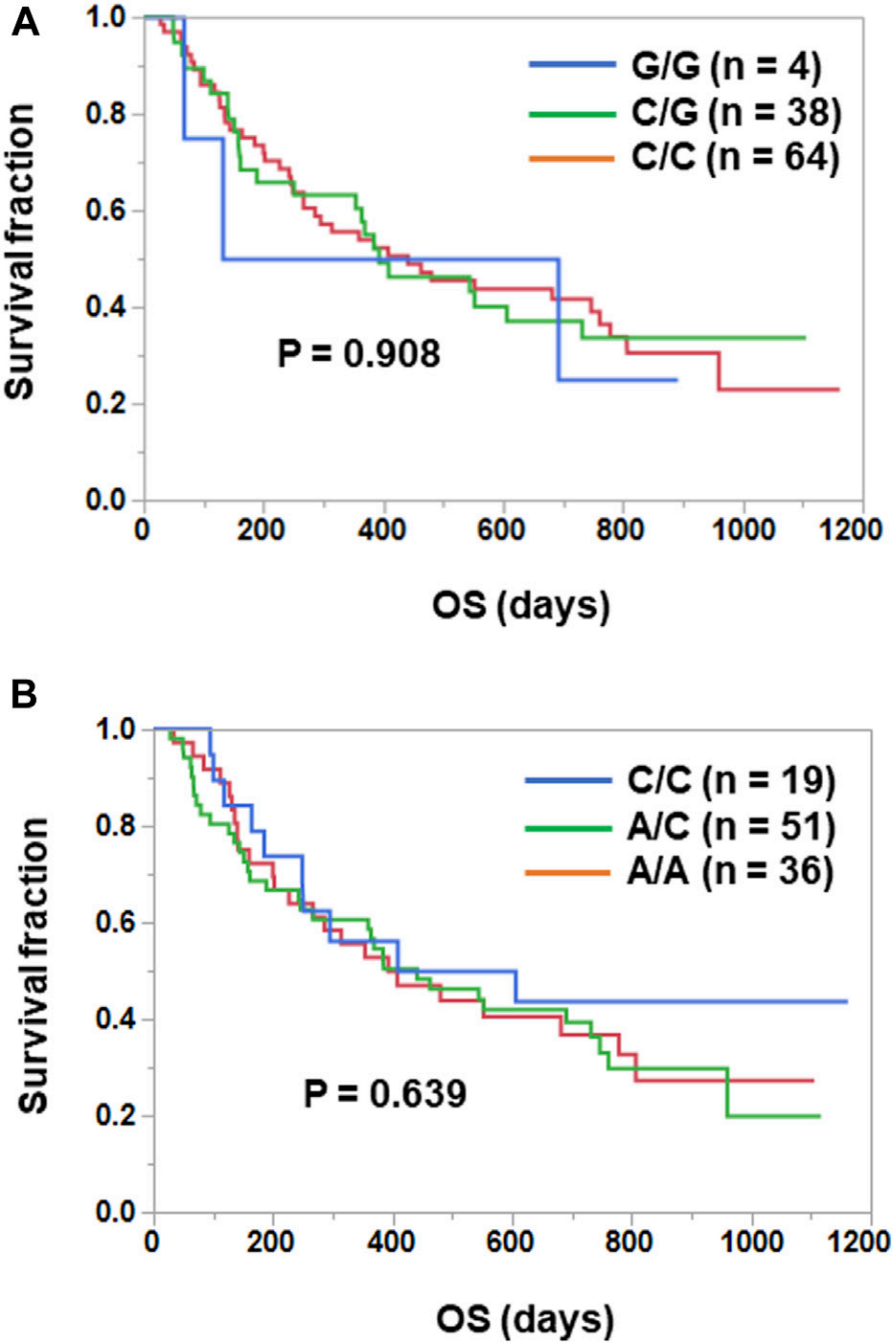
No prognostic significance of IL-6 (−634G>C) and IL-6R (48892A>C) genetic polymorphisms in NSCLC patients treated with ICIs. **(A)** Patients treated with ICIs were divided into three subgroups according to the IL-6 (−634G>C) polymorphism [IL-6 -634G/G (*n* = 4), C/G (*n* = 38), C/C (*n* = 64)]. Curves for OS were estimated by the Kaplan-Meier method and statistically evaluated by the log-rank test (*p* = 0.908). **(B)** Patients treated with ICIs were divided into three subgroups according to the IL-6R 48892A>C polymorphism [IL-6R 48892C/C (*n* = 19), A/C (n = 51), A/A (*n* = 36)]. Curves for OS were estimated by the Kaplan-Meier method and statistically evaluated by the log-rank test (*p* = 0.639).

**FIGURE 3:**
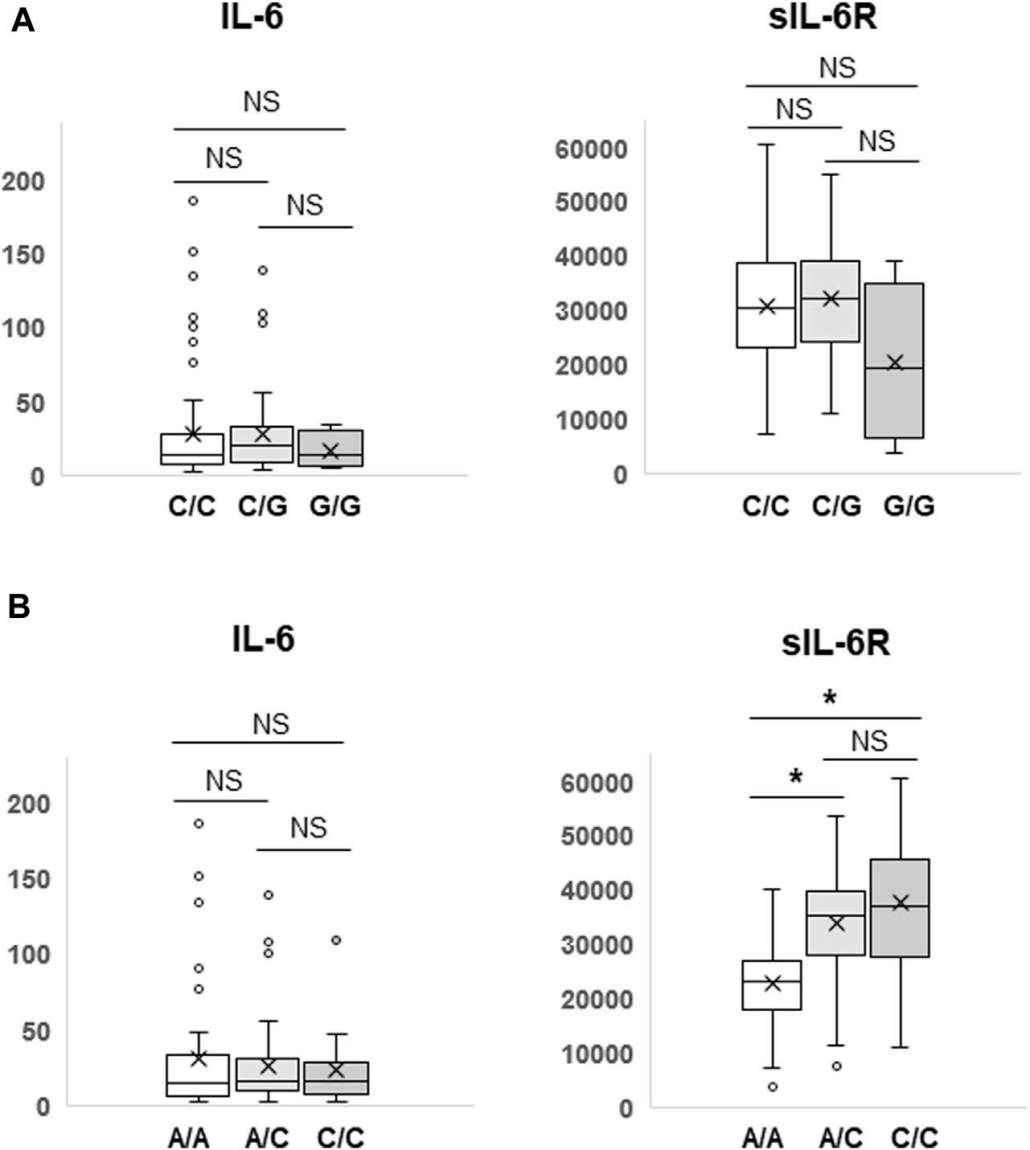
Differences in plasma IL-6 and sIL-6R levels according to the IL-6 (−634G>C) or IL-6R (48892A>C) polymorphism. Plasma IL-6 and sIL-6R levels were compared among three subgroups according to the IL-6 (−634G>C) **(A)** or IL-6R (48892A>C) **(B)** polymorphism by Student’s t-test. Data are presented as box plots. The bottom and top of the box represent the first and third quartiles, and the band and “X” inside the box correspond to the median and mean, respectively. Whiskers indicate the variability outside the upper and lower quartiles. Outliers are shown as individual points. *p < 0.001. NS, not significant (*p* > 0.05).

IL-6 or IL-6R genetic polymorphisms have been reported to be associated with risk or prognosis in patients with some cancers (DeMichele et al., 2009; Motoyama et al., 2012; Stephens et al., 2012; Kibe et al., 2014; Ruzzo et al., 2014; Zhang et al., 2017; Gu et al., 2023). Therefore, we investigated the clinical significance of genetic polymorphisms of IL-6 (−634G>C) and IL-6R (48892A>C) in NSCLC patients treated with ICIs. As shown in Figure 2, Kaplan-Meier curves showed no significant separation among subgroups stratified by the IL-6 (*p* = 0.908) or IL-6R (*p* = 0.639) genetic polymorphism. Furthermore, the IL-6 634G>C polymorphism had no effect on plasma IL-6 (C/C vs. C/G, *p* = 0.964; C/C vs. G/G, *p* = 0.220; C/G vs. G/G, *p* = 0.215) and sIL-6R (C/C vs. C/G, *p* = 0.517; C/C vs. G/G, *p* = 0.254; C/G vs. G/G, *p* = 0.207) levels (Figure 3A). However, as expected (Zhang et al., 2022; Rose-John et al., 2023), the IL-6R 48892A>C polymorphism showed a significant effect on plasma sIL-6R (A/A vs. A/C, *p* < 0.001; A/A vs. C/C, *p* < 0.001; A/C vs. C/C, *p* = 0.222), but not IL-6 (A/A vs. A/C, *p* = 0.581; A/A vs. C/C, *p* = 0.432; A/C vs. C/C, *p* = 0.703) levels (Figure 3B).

## 4 Discussion

There have been several reports on the prognostic significance of plasma IL-6 in ICI-treated patients with various cancers, including NSCLC (Kang et al., 2020; Keegan et al., 2020; Laino et al., 2020; Kauffmann-Guerrero et al., 2021; Liu et al., 2022; Wang et al., 2022; Hu et al., 2023; Inoue et al., 2023); however, the clinical significance of its receptors, sIL-6R or sgp130, in the plasma has not been clarified. This is the first study to investigate the clinical significance of plasma sIL-6R and sgp130 levels and the ratios of binary IL-6:sIL-6R and ternary IL-6:sIL-6R:sgp130 complexes in ICI-treated NSCLC patients. Our results showed that plasma IL-6 levels before ICI treatment were significantly associated with OS, whereas plasma sIL-6R and sgp130 levels showed no prognostic significance. Furthermore, the ratios of binary IL-6:sIL-6R and ternary IL-6:sIL-6R:sgp130 complexes were not associated with OS.

IL-6 is a multifunctional cytokine that regulates various aspects of the immune response and acute phase response (Hunter and Jones, 2015; Johnson et al., 2018; Jones and Jenkins, 2018). In addition, IL-6 has been reported to be one of the critical cytokines for the induction of suppressive immune cell subsets, such as myeloid-derived suppressor cells and Th17, which are known to negatively affect anti-tumor immunity (Hunter and Jones, 2015; Johnson et al., 2018; Jones and Jenkins, 2018). Thus, it is possible that high levels of IL-6 inhibit immune responses induced by ICI treatment. Indeed, in murine tumor models, treatment combined with ICI and IL-6 blockade was reported to lead to increased tumor shrinkage *in vivo*, with a higher density of CD4^+^/CD8^+^ effector T cells and reduction of Th17, macrophages, and myeloid cells within tumor tissues (Hailemichael et al., 2022). IL-6 has been reported to coordinate inflammatory responses in a context-dependent manner by triggering two distinct signaling modes, classical signaling and trans-signaling (Rose-John et al., 2006; Garbers et al., 2011; Rose-John et al., 2023). In the classical signaling, IL-6 binds directly to the membrane-bound IL-6R expressed on specific cells, such as hepatocytes, monocytes, or macrophages, which, together with IL-6, binds to and induces the dimerization of a second receptor subunit, gp130. In contrast, in the trans-signaling, IL-6 binds to sIL-6R and forms an IL-6:sIL-6R complex that interacts with gp130, which is expressed on almost all cell types regardless of IL-6R expression. However, the IL-6:sIL-6R complex is neutralized by sgp130 forming the ternary IL-6:sIL-6R:sgp130 complex. Therefore, the level of IL-6 trans-signaling depends on the level of sIL-6R and the neutralizing capacity of sgp130 (Gaillard et al., 1999; Garbers et al., 2011). Previous studies have suggested that circulating levels of sIL-6-R and sgp130 are associated with the risk of inflammation-related diseases, such as CVE and metabolic syndrome (Weiss et al., 2013; Moreno Velasquez et al., 2015; Ritschel et al., 2016). In addition, the ratios of the IL-6:sIL-6R and IL-6:sIL-6R:sgp130 complexes have been reported to reflect the level of IL-6 trans-signaling and influence the risk or severity of inflammation-related diseases, such as CVE and COVID-19 infection (Ziegler et al., 2019; Miri et al., 2021; Rodriguez-Hernandez et al., 2022; Li et al., 2023). However, in this study, neither plasma sIL-6R and sgp130 levels nor the ratios of IL-6:sIL-6R and IL-6:sIL-6R:sgp130 complexes showed prognostic significance, suggesting that the IL-6 classical signaling, but not trans-signaling, may be related to anti-tumor immune responses in cancer patients treated with ICIs.

In addition to the IL-6 classical signaling and trans-signaling described above, Heink et al., 2017 recently demonstrated a third mode of IL-6 signaling, termed IL-6 cluster signaling (Schumertl et al., 2022). In this mode, IL-6 is bound to the IL-6R on dendritic cells (DCs) and trans-presented to T cells, which are then activated via gp130 homodimerization. This IL-6 cluster signaling has been reported to be crucial for the development of pathogenic Th17 cells, and depletion of IL-6 or IL-6R only on DCs protected mice in an experimental autoimmune mouse model (Heink et al., 2017). Since Th17 cells have also been reported to be highly associated with anti-tumor immunity, IL-6 cluster signaling may be related to anti-tumor immune responses in cancer patients treated with ICIs. Future studies are warranted to evaluate a potential impact of this third mode on anti-tumor immunity.

It has been demonstrated that IL-6-mediated signaling can be inhibited by three different approaches at the ligand and/or receptor level; direct targeting of IL-6 or IL-6R with Abs and targeting of the IL-6:sIL-6R complex with sgp130 fusion proteins (Johnson et al., 2018; Jones and Jenkins, 2018; Rose-John et al., 2023). Anti-IL-6 Ab, such as siltuximab, and anti-IL-6R Ab, such as tocilizumab, have been reported to inhibit both classical signaling and trans-signaling, and their effects have been investigated in various cancer types in preclinical and early-stage clinical studies (Jones and Jenkins, 2018; Rose-John et al., 2023). In contrast, sgp130 fusion proteins, such as olamkicept, have been reported to selectively inhibit trans-signaling, and have shown promising results in clinical trials for rheumatoid arthritis and inflammatory bowel diseases (Jones and Jenkins, 2018; Rose-John et al., 2023). Because our results suggested that the IL-6 classical signaling, but not trans-signaling, may be related to the anti-tumor immune responses in NSCLC patients treated with ICIs, anti-IL-6 or anti-IL-6R Abs, but not sgp130 fusion proteins, may be promising to improve the efficacy of ICIs. Indeed, combined ICI and anti-IL-6 or anti-IL-6R Abs have been reported to potentially enhance anti-tumor immunity or reduce immune-related toxicity in preclinical (Mace et al., 2018; Hailemichael et al., 2022) and clinical studies (Fa’ak et al., 2023). Therefore, to facilitate personalized immunotherapy, further clinical trials are recommended to validate the effects of anti-IL-6 or anti-IL-6R Abs in ICI-treated NSCLC patients with high plasma IL-6 levels.

It has been reported that IL-6 or IL-6R genetic polymorphisms are associated with risk or prognosis in patients with some cancers (DeMichele et al., 2009; Motoyama et al., 2012; Stephens et al., 2012; Kibe et al., 2014; Ruzzo et al., 2014; Zhang et al., 2017; Gu et al., 2023). For example, our previous study demonstrated that the IL-6 -634G>C genetic polymorphism was associated with prognosis after surgery in advanced thoracic esophageal squamous cell carcinoma (Motoyama et al., 2012). In addition, the IL-6 -634G allele was reported to decrease the risk of high-grade irAEs in patients with solid tumors receiving ICIs (Xin et al., 2023). However, in this study, this polymorphism had no effect on prognosis in ICI-treated NSCLC patients. The IL-6R 48892A>C genetic polymorphism has been shown to result in a change from Asp358 to Ala358 near the ADAM17 cleavage site of the IL-6R protein (Zhang et al., 2022; Rose-John et al., 2023). Indeed, the Ala358 variant of the IL-6R resulting from IL-6R 48892A/C or C/C genotypes shows a more efficient cleavage of the membrane-bound IL-6R, resulting in a reduced number of functional membrane-bound IL-6R, accompanied by higher levels of circulating sIL-6R in the blood. Consequently, carriers of the Ala358 variant of the IL-6R were shown to be less susceptible to several inflammatory diseases, such as congestive heart disease, abdominal aortic aneurysm, and rheumatoid arthritis (Zhang et al., 2022; Rose-John et al., 2023), possibly due to attenuation of classical IL-6 signaling by reduction of the membrane-bound IL-6R on the surface of target cells. We also demonstrated that the IL-6R A/C or C/C genotype showed a prolonged OS in colorectal cancer patients who received cancer vaccination (Kibe et al., 2014). In contrast, the IL-6R 48892 C/C genotype was reported to have an adverse prognostic effect in patients with advanced gastric cancer (Ruzzo et al., 2014). However, in the current study, although this polymorphism significantly affected the levels of circulating sIL-6R levels in the plasma as expected, it had no prognostic significance in ICI-treated NSCLC patients. Nevertheless, further studies are needed to clearly define the relationship between the genetic polymorphisms and anti-tumor immunity.

In conclusion, we demonstrated that plasma levels of IL-6, but not of its receptors sIL-6R and sgp130, were associated with OS in NSCLC patients receiving ICI therapy. However, this study had limitations. In particular, this study included patients with different clinical characteristics, such as PS, driver mutations, PD-L1 expression in tumor tissue, and type of PD-1/PD-L1 inhibitors, but the number of patients was relatively small to perform subanalyses. Given the growing interest in the factors related to the clinical efficacy and mechanisms of action of ICIs, further large-scale patient studies are warranted to provide more detailed analyses and increase the validity and generalizability of our findings. We are currently working to increase the sample size for our research by expanding our recruitment efforts and collaborating with other institutions, which will provide more robust results in future studies.

## Data Availability

The raw data supporting the conclusion of this article will be made available by the authors, without undue reservation.
